# Mental health policy and practice – second edition

**Published:** 2015-09-21

**Authors:** Charlotte Klinga

**Affiliations:** Medical Management Centre, Karolinska Institutet, Stockholm, Sweden Email: charlotte.klinga@ki.se

This book takes you on a journey through key changes and development of mental health policy and practice in the UK. By combining case studies, reflections and analysis followed by exercises and examples of further reading, the evolution of contemporary mental health services is described. As a reader you become acquainted with the rationale behind the path of the current policy and implications for future practice. The book is a revised version of the first edition which was published in 2006.

The book includes 11 chapters of which the last one is a conclusion. Each chapter starts off with an overview of themes to be discussed and ends up with suggestions for further reading. The number of pages in each chapter is between 16 and 35. The book also contains an appendix of mental health policy chronology (1975–2008) and an overview of selected key legislation.

Chapter 1 explains what the authors perceive as mental health, the strategic importance of mental health and different ways of thinking about mental health. The incidence and prevalence of mental health problems are deliberated upon, then follow a review of the book and finally a summary of the key themes of each chapter including the authors’ reflections upon the mental health system.

Chapter 2 gives the reader an overview of the mental health policy. In order to understand the rest of the book, the authors believe that this might be helpful. I do agree. Those readers of the book, who wish to obtain a detailed chronological history of mental health policy in the UK, will find this chapter pleasant. I found the last part about reasons why policies are not always implemented in practice as very an interesting as well as the summary of frameworks aiming at explaining current policy tensions and dilemmas.

Chapters 3, 4, 5 and 6 discuss in the following order the service areas: primary care, community mental health, acute care and forensic mental health services. The chapters do not only give an extensive overview of the structure, origin, role and trends of the different service areas, they also stress key concerns and suggestions from the authors of what is needed to provide high quality care for people with mental health problems. Each chapter contains several thought-provoking parts. In chapter 3, for instance, a discussion of integrated models of working in primary care mental health settings is included and in chapter 4 there is put emphasis on alternative ways of providing community-based services and examples of positive practice in community inclusion. In chapter 5 the role of national guidance to improve the physical environment of acute wards is stressed and the development of a more generalisable model of care which is experienced as safe and therapeutic by the users. Lastly, in chapter 6 an important and highly relevant discussion about the ‘mad versus bad’ debate can be found. Misunderstandings about the alleged link between mental illness and crime/violence are sorted out.

Chapters 7, 8, 9, 10 provide a broader picture of the mental health policy and practice in the UK by addressing several important aspects, including partnership working, user involvement, anti-discriminatory practice and carers’ perspective. Especially chapter 7 is interesting: it lifts the burning question concerning the lack of co-ordinated services, not only in the UK but also in most other western countries. How does the fragmented care impact the quality and safety of the care that mental health care users receive? This discussion ties in with the next chapter's discussion of the view of mental health service users through the years, as well as the history of user involvement. Both barriers and examples of positive examples are highlighted. In chapter 9, the reader gets acquainted with the concepts of discrimination including current policy context and relevant legislation. Particularly highlighted is the discrimination of different groups such as minority ethnic communities, people with physical impairments or learning difficulties, LGBT persons. Chapter 10 focuses on the important role of carers, their expertise and contribution. Similarly their own needs are discussed. I am inclined to agree with the authors who believe that interpersonal skills and human skills such as ability to listen and empathy needs to be more valued and acknowledged.

Chapter 11 ends up with a short, but nonetheless, very interesting and thoughtful discussion about the rhetoric/reality gap between policy and practice. Key themes are extracted by the authors and underlying reasons are suggested. As a final point the authors leave a couple of key questions lacking shared answers. They argue that answers can only be received by listening more fully to the voices of mental health service users, their families and mental health service workers.

To conclude this book origin in a systems approach, emphasising the importance of understanding and learning about mental health from various perspectives in order to develop mental health services which can better meet the needs of the service users, their families and the society as a whole. The authors have not taken any political or theoretical stance. They discuss the topics from different perspectives, some of which are contradictory. One central theme throughout the book is the need of service user involvement together with increased cooperation, coordination and collaboration. The book is pedagogically well designed and therefore very reader friendly. It can be read from cover to cover if you wish to receive a more coherent picture of the mental health policy development in the UK. However, it works just as well to let your interest in the different topics guide the order in which you read the chapters of the book. This book is mainly for professionals working within the field of mental health and social care and students planning to work within this field. However, this book can also be valuable for policy makers and managers in the health and social care sector.

